# Effects of arsenic trioxide, all-trans-retinoic acid and dexamethasone on NB4 cell line

**Published:** 2010

**Authors:** A. Mandegary, M. Mehrabani

**Affiliations:** 1 Pharmaceutics Research Center and Department of Toxicology and Pharmacology, Faculty of Pharmacy; 2 Central Laboratory, Deputy of Research; 3 Department of Pharmacogenosy, Faculty of Pharmacy, Kerman University of Medical Sciences, Kerman, Iran

**Keywords:** ATO, ATRA, Dexamethasone, Combination therapy, Apoptosis.

## Abstract

**Background and the purpose of the study:**

Experimental and preclinical observations have indicated that combination therapy with all-trans-retinoic acid (ATRA) and arsenic trioxide (ATO) may strongly enhance their therapeutic effects in the treatment of acute promyelocytic leukemia (APL). Whilst dexamethasone (Dex) is routinely used for the control of APL- differentiation syndrome, its effect on the pharmacodynamics of ATO is not clear. Therefore, in this study, effects of therapeutic concentrations of ATO, ATRA and Dex and their sequential usages on the proliferation, differentiation and apoptosis in t(15;17)-positive NB4 cells was investigated.

**Methods:**

Cells were treated with therapeutic concentrations of ATO, ATRA and Dex either as single or in combination and cell proliferation was assessed by XTT assay. Expression of CD11b as an indicator of cell differentiation and the percentage of 7-AAD positive cells as a marker of apoptosis were determined by flow cytometry.

**Results:**

ATO, but not ATRA and Dex, decreased proliferation of the cells dose-dependently. Pre-treatment of the cells with any of the drugs did not alter the effects of other drugs on the proliferation. Pre-treatments with Dex blocked the apoptotic effect of ATO (1 µM).

**Conclusion:**

No improvement or antagonistic effects was observed with the pretreatment/ combination of the ATO and ATRA on the differentiation and apoptosis of the cells. It is possible that concomitant usage of Dex with apoptotic doses of ATO in APL patients counteract therapeutic effects of ATO.

## INTRODUCTION

Acute myeloid leukemia (AML) is a disease characterised by abnormalities in the proliferation/ differentiation coupling. The exact cause of leukemia is not yet well known; however, family history, smoking, long term exposure to chemicals such as benzene, prolonged exposure to radiation (1) and abnormalities in the processes of cell proliferation, differentiation and apoptosis (2–4) have been mentioned for this disease. Acute promyelocytic leukemia (APL) is a well-defined subtype of leukemia which accounts for 10-15% of acute myeloid leukemias. The APL is characterized by PML/RARα fusion protein resulting from a reciprocal translocation between the retinoic acid receptor-α (RARα) gene on the chromosome 17 and the PML gene on the chromosome 15. The PML/ RARα is leukemogenic and blocks the differentiation and apoptosis of promyelocytes. All-trans retinoic acid (ATRA) has therapeutic effect in APL due to its capability to induce the differentiation of leukaemia cells into mature granulocytes.

Recently, arsenic trioxide (ATO) has been used to treat relapsed and newly diagnosed APL patients (5, 6). At the molecular level, ATO exerts a number of dose-dependent dual effects on APL cells such as induction of apoptosis and partial differentiation at concentrations of 0.5-2.0 µM and 0.1-0.5 µM, respectively (7). Recently we have shown the role of Bax, p38 and ERK1 proteins in differentiation and apoptosis of promyelocytes in APL patients during treatment with ATO (6). There are controversial in vivo and in vitro reports on the preference of ATRA/ATO combination therapy for APL and non- APL over monotherapy with each of them (5, 8–10). Also there are contradictory reports on in vitro pre-treatment and combination therapy of ATO and ATRA (8, 11).

APL-differentiation syndrome is a life threatening adverse effect of differentiating agents like ATO and ATRA used for the treatment of APL. Administration of high doses of corticosteroids especially Dex have proved to be very effective treatment for prevention of this syndrome (6, 12). Highdoses of methylprednisolone has been shown to induce in vivo and in vitro differentiation of myeloid leukemia cells to mature granulocytes in patients with APL and other subtypes of AML (13). However, an important question is whether the prophylactic and concomitant corticosteroid treatment could potentially affect the efficacy and potency of ATO and ATRA therapy. This study was undertaken to delineate the possible interferences of Dex with the therapeutic actions of ATO and ATRA to address the aforementioned question.

In this study effects of pre-treatment with ATRA and Dex on ATO-induced apoptosis, effects of Dex on ATO and ATRA-induced differentiation and effects of combination of these drugs on proliferation and/or toxicity of NB4-APL cell line were investigated.

## MATERIAL AND METHODS

### 

#### Reagents

Arsenic trioxide (As_2_O_3_; ATO) was purchased from Sigma Chemical Co (St Louis, MO, USA) and dissolved in NaOH alkalinized deionized water in the stock concentration of 1 M and then diluted with PBS to a working concentration of 50 µM. All- trans retinoic acid was purchased from Sigma and dissolved in 100% ethanol to a stock concentration of 1 M, stored light protected at -20°C. For every experiment the fresh working solution of 500 µM of ATRA was prepared in absolute ethanol. Water soluble Dex (Dexamethasone dihydrogenphosphate; Dexa-rattiopharm®, Germany) was diluted to working solution of 500 µM with phosphate-buffered saline (PBS, 137 mM NaCl, 2.7 mM KCl, 10 mM Na_2_HPO_4_, 2 mM KH_2_PO_4_, pH of 7.4).

#### Cell culture

The NB4 promyelocytic cell line was maintained in complete medium containing RPMI 1640 medium (Sigma, USA) with 10% FBS (Gibco, USA), 100 U/ml penicillin and 100 mg/ml streptomycin (Gibco, USA) in a humidified atmosphere with 5% CO at the concentration of 1×10^5^/ml-1×10^6^/ml cells.

#### Pre-treatment and treatment of cells

For pre-treatment, cells were cultured in four groups using complete medium in 25 cm2 tissue culture flasks. The first group was pre-treated with ATO (0.25 µM), the second with ATRA (1 µM), the third with Dex (1 µM) and the forth with no pre- treatment. After 24 hrs, all groups of cells were washed with PBS and re-suspended in complete medium containing the same reagents as above. Then, all cells were subjected to further treatments as follows: Group 1 was divided in three subgroups receiving ATRA (0.5 and 1 µM respectively) and 1 µM of Dex; group 2 was divided in three subgroups receiving ATO (0.25 and 1 µM respectively) and 1 µM of Dex; three subgroups of group 3 received ATO (0.25 and 1 µM) and 1 µM of ATRA respectively. Group 4 was divided in four subgroups receiving ATRA (0.5 and 1 µM respectively), 0.25 µM of ATO and 1 µM of Dex.

In co-treatment experiments, each of the four main groups of cells were each treated for 24 hrs with one main drug and various concentrations of other drug partners as outlined in the legends of respective graphs.

#### XTT assay for cellular proliferation

Aliquots of 100 µl of the cell suspension containing 30,000 cells were dispensed into 96-well flat- bottomed microplates. The proliferation of the cells was assessed using XTT Cell Proliferation Kit II (Roche, Germany) according to the manufacturer's guideline. Eexperiments were performed in triplicates and repeated three times. Absorbance at 450 nm (A_450nm_) of the formazan was measured using a microplate reader adjusted to zero by the reagent blanks to correlate the colored product with the number of cells, and the results were expressed as a ratio of the treated cells over the untreated cells.

#### Flow cytometric analysis of differentiation

Cells were incubated for 30 min with PE- conjugated mouse anti-human CD11b antibody (Becton Dickinson, USA). Control studies were performed with non-binding control mouse IgG2a isotype antibodies (Becton Dickinson, USA). The percentage of CD11b positive cells was determined by measurement of the fluorescence intensity using FAC Scan (Becton Dickinson, USA)

#### Apoptosis assay

For staining of apoptotic and dead cells, the 7- amino-actinomycin D (7-AAD) method was used (14). Briefly, after culture of NB4 cells to 24-well plate, the cells were incubated with ATO (0.25 µM), ATRA (1 µM) or Dex (1µM). After 24 hrs incubation in a CO_2_ incubator, the second drug (ATO, ATRA or Dex) was added to the corresponding well, then after 6, 12 and 24 hrs an aliquot of cells was harvested by centrifugation and incubated with 20 µg of 7- AAD per ml in PBS containing 2% calf serum and 0.1% sodium azide (Sigma, USA) (PBSAz) and in the absence of Ca^2+^ and Mg^2+^, for 20 min at 4°C protected from light. The cells were then analyzed on a FACScan flow cytometer (Becton Dickinson) in the manufacturer's staining solution. All data were collected, stored and analyzed by Lysis II software (Becton Dickinson, USA).

#### Statistical analyses

Data were expressed as Means ± SEM of percentage of control. One-way analysis of variance (ANOVA) followed by the Tukey HSD was used to assess significant differences between treatment groups. Differences were considered as significant when p<0.05.

## RESULTS

### 

#### Effects of ATO, ATRA and Dex on proliferation of NB4 cells

ATO decreased the proliferation of NB4 cells significantly (p<0.001) at the concentration of 1 µM. In contrast, ATRA (0.5 and 1 µM) and Dex (1 µM) only increased the proliferation of NB4 cells insignificantly.

#### Effects of combination treatment with ATRA and Dex on the antiproliferative activity of ATO

**Figure 1:**
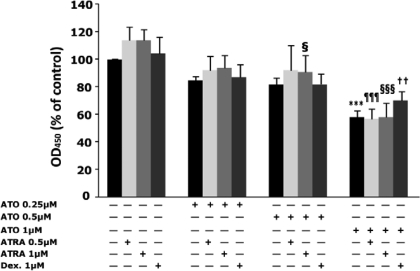
Proliferation of NB4 cells in response to therapeutic concentrations of ATO, ATRA and Dex alone or in combination. Cell proliferation was assessed by XTT assay for cellular proliferation. *** p< 0.001 compared to NB4 without any treatment, ¶¶¶ p<0.001 compared to ATRA at 0.5 µM, § p<0.05 and §§§ p<0.001 compared to ATRA at 1 µM and †† p>0.01 compared to Dex, at 1 µM, One-way ANOVA.

The antiproliferative profile of ATO in concentrations of 0.25 and 0.5 µM was not changed when ATO used with therapeutic concentrations of ATRA (0.5 and 1 µM) and Dex (1 µM) (Fig 1). Combined treatment of Dex with 1 µM of ATO counteracted the antiproliferative effect of ATO, but not significantly.

#### Effects of pre-treatment of NB4 cells with ATO on the anti-proliferation of cells by ATRA and Dex

**Figure 2:**
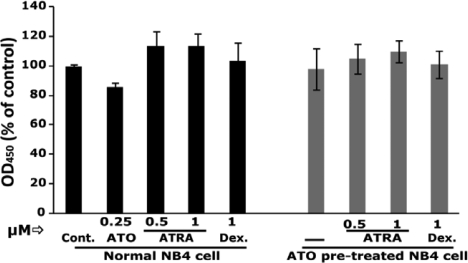
Proliferation of pre-treated NB4 cells with 0.25 µM of ATO in response to therapeutic concentrations of ATRA and Dex. The cells were pre-treated with 0.25 µM of ATO for 24 hrs and then second drugs namely ATRA and Dex were added to cells. Normal NB4 cells were not pre-treated with ATO.

Figure 2 shows that the antiproliferative profile of ATRA (0.5 and 1 µM) and Dex (1 µM) didn't change when the NB4 cells were pre-treated with low concentration of ATO (0.25 µM).

#### Effects of Dex pre-treatment on the anti-proliferation of cells by ATO

**Figure 3:**
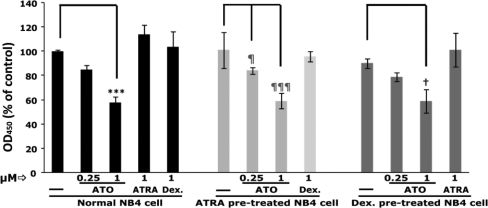
Effect of ATRA and Dex pre-treatment on the proliferation of NB4 cells in response to ATO, ATRA and Dex. NB4 cells pre-incubated with ATRA (1 µM) and 1 µM Dex 24 hrs before addition of the second drug. Cell proliferation was assessed by XTT assay for cellular proliferation. *** p< 0.001 compared to normal NB4 and ¶ p<0.05, ¶¶ p<0.01 compared to ATRA pre-treated cells and † p<0.05 compared to Dex. Pre-treated cells, One-way ANOVA.

Pre-teatment of NB4 cells with 1 µM of ATRA did not change the anti-proliferative effects of ATO (0.25 and 1 µM) and 1 µM of Dex (Fig 3). Pre- treatment with 1 µM of Dex also didn't change the anti-proliferative effects of ATO and ATRA at concentration of 1 µM (Fig 3).

#### Effects of Dex on differentiation of NB4 cells by ATRA

**Figure 4:**
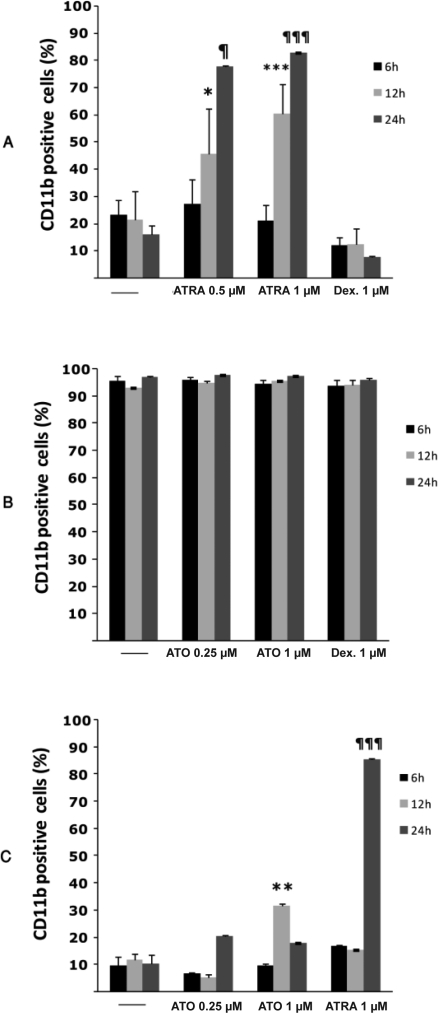
Effects of pre-treatment on the CD11b cell surface expression of NB4 cells after exposure to ATO, ATRA and Dex. NB4 cells were treated first with ATO (0.25 µM), ATRA (1 µM) and Dex (1 µM) for 24 hrs and then second drug was added to cells. **(A)** Effects of ATRA and Dex on the expression of CD11b in the cells pre-treated with ATO. **(B)** Effects of ATO and Dex on the expression of CD11b in the cells pre-treated with ATRA. **(C)** Effects of ATO and ATRA on the expression of CD11b in the cells pre-treated with Dex. Flow cytometric analysis for CD11b cell surface expression was carried out after 6, 12 and 24 hrs. * p< 0.05, ** p< 0.01 and *** p< 0.001 in comparison to control of each pretreatment using One-Way ANOVA.

Pre-treatment with ATO at 0.25 µM induced differentiation to nearly 25% in NB4 cells as measured by expression of the CD11b marker. Addition of ATRA to the cells induced CD11b expression to 100% after 24 hrs but Dex insignificantly decreased CD11b expression (Fig 4). ATRA induced the expression of CD11b, which its pattern of expression was unaffected by ATO or Dex. CD11b expression was low (10%) with Dex and its presence didn't counteract CD11b expression after addition of ATO and ATRA.

#### Effects of pre-treatment with Dex on the apoptotic effects of ATO and ATRA

**Figure 5:**
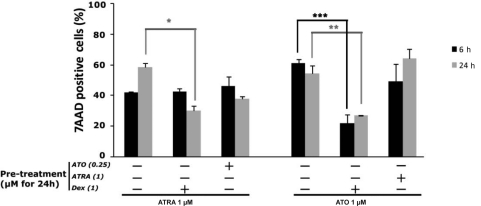
Effects of pre-treatment of NB4 cells with ATO, ATRA and Dex on apoptotic effects of ATO and ATRA. NB4 cells were treated with ATO (0.25 µM), ATRA (1 µM) and Dex (1 µM) for 24 hrs. Effects of 1 µM of ATRA on the ATO and Dex pre-treated cells are shown at left. The apoptotic effects of ATO at 1 µM on the ATRA and Dex pre-treated cells are shown in right. Flow cytometric analysis for CD11b cell surface expression was carried out after 6 and 24 hrs. * p< 0.05, ** p< .01 and *** p< 0.001 using One-Way ANOVA.

NB4 cells were pre-treated with 1 µM of Dex, 0.25 µM of ATO or 1 µM of ATRA for 24 hrs before exposure to 1 µM of ATO or 1 µM of ATRA. The results showed that pre-treatment with Dex could counteract the apoptotic effects of ATRA and ATO (Fig 5). These effects were even maintained after withdrawal of each pre-treatment (data are not shown). Meanwhile pre-treatment with ATO did not change the apoptotic effect of ATRA and vice versa.

## DISCUSSION

There are contradictory reports about the potential advantages of ATRA ATO combination therapy for APL over monotherapy with each of them (5, 8–10, 15). Gianni et al (8) have reported that combination therapy with ATRA ATO gives better results than therapy with either of the two drugs in terms of complete remission and status of the disease- free survival. However ATRA did not improve the response to ATO in relapsed APL patients in another study (9). Also in agreement with Gianni's report, better results for ATRA/ATO combination therapy than monotherapy has been reported (15). On the other hand, Ghavamzadeh et al (5) have shown that ATO, with or without ATRA, can yield complete remission rate of 86.3% in the newly diagnosed and relapsed APL patients. Recently, Ravandi et al (10) have concluded that combination of ATO and ATRA as first line therapy for APL was effective and safe. The finding that ATRA promotes in vivo ATO- dependent efficacy, prompted us to perform further studies to define whether the sequence of these drugs’ administration influence their therapeutic effects, namely induction of differentiation and apoptosis. In addition, the effect of Dex, which is typically used to control the symptoms of differentiation of APL cells to mature cells, on the effects of ATO and ATRA treatment in the NB4 cells were investigated. In general aim of this study was to determine if there were any effects that could potentially have impact on safety and efficacy of this treatment regimen. Treatment of NB4 cells with therapeutic concentrations of ATO (0.5-1 µM), ATRA (1 µM) and Dex (1 µM) for 24 hrs showed that only ATO decreased the proliferation of NB4 cells in a dose- dependent manner leading to significant changes at 1 µM concentration. Conversely, ATRA and Dex increased the proliferation of cells after 24 hrs to an insignificant level (Fig 1).

Results of the evaluation of pre-treatment efficacy showed that the proliferation of pre-treated NB4 cells did not change under the effect of the second added drug as compared to unpretreated cells (Figs 2 and 3).

It was also investigated whether pre-treatment with ATO, ATRA and Dex would interfere with differentiation induced by each of them separately. ATO at 0.25 µM slightly increased differentiation of NB4 cells but did not potentiate the expression of CD11b induced by ATRA (Fig 4-A). Dex by itself neither induced NB4 cell differentiation nor affected the immunophenotypic parameter associated with the granulocytic differentiation induced by ATO and ATRA (Fig 4-C). This finding does not support the reports on the inductive effects of Dex on differentiation of acute myeloid leukemia and APL (16).

To evaluate the effect of pre-treatment/combination therapy on the induction of apoptosis, after treatment of cells similar to previous experiments, frequency of apoptotic cells were assessed.

The observation that ATO-induced apoptosis in NB4 cells was not changed by pre-treatment with ATRA (Fig 5) is consistent with the result of experiments published by Chen et al (17) and Gianni et al (8). However, Jing et al (11) have shown that pre-treatment with ATRA decreased ATO-induced apoptosis in ATRA-sensitive NB4 but not ATRA-resistant cells. Notably, pre- treatment of cells with Dex significantly decreased the apoptosis induced by 1 µM of any of ATO and ATRA (Fig 5). Evidences demonstrate that ATO and ATRA exert some of their anti-leukemic effects through production of reactive oxygene species (ROS) (18, 19). NADPH oxidase has a major role in arsenic and ATRA-induced reactive oxygen species production, cytotoxicity and maturation of APL cells (18, 20). Condino-Neto et al (21) have shown that Dex down-regulates the components of NADPH oxidase and their activities. Therefore one can translate the suppressive effect of Dex on the apoptotic effects of ATO and ATRA to the decrease in the activity of NADPH oxidase.

Taken together, results of this study showed that pre-treatment of NB4 cells with therapeutic concentrations of ATO and ATRA did not improve the differentiation and apoptotic effects of each of them. Moreover, using Dex with higher concentrations of ATO may counteract therapeutic effects of ATO.
